# Effect of Acute and Chronic Oral Zinc Administration in Hyperprolactinemic Patients

**DOI:** 10.1155/MBD.1999.159

**Published:** 1999

**Authors:** Guiomar Madureira, Walter Bloise, Berenice Bilharinho Mendonca, José Brandão-Neto

**Affiliations:** 1 Division of Endocrinology and Metabolism Department of Internal Medicine Universidade de São Paulo São Paulo Brazil; 2 Division of Endocrinology and Metabolism Department of Internal Medicine Universidade de Brasília – DF CP 4591 Brasília DF CEP 70919-970 Brazil

## Abstract

The inverse relationship between zinc (Zn^++^) and prolactin (PRL) was detected in *in vitro* studies, whereas *in vivo* results are contradictory. In order to evaluate this controversial subject we studied patients with hyperprolactinemia. Basal serum Zn^++^ levels and serum PRL response to acute and chronic oral Zn^++^ administration were evaluated in seven patients with prolactinomas and one with idiopathic hyperprolactinemia. Serum PRL levels did not change after acute oral Zn^++^ administration (37.5 mg), although Zn^++^ levels increased from 1.11±0.15 to 2.44±0.39 μg/mL (P<0.05). ZnZn^++^ administration (47.7 mg daily) during 60 days increased serum Zn^++^ levels from 1.11 ± 0.15 to 1.59 ± 0.58 μg/mL (*p* < 0.05) but caused no change in serum PRL levels. The TRH tolerance test (200 μg) was performed before and after 60 days of Zn^++^ administration, and PRL response to TRH was unchangeable and similar in both tests. We concluded that acute or chronic Zn^++^ administration does not inhibit PRL secretion in basal condition or by TRH effect in hyperprolactinemic patients.


### EFFECT OF ACUTE AND CHRONIC ORAL ZINC ADMINISTRATION IN HYPERPROLACTINEMIC PATIENTS

Guiomar Madureira[1], Walter Bloise[1], Berenice Bilharinho Mendonca[1], and José Brandão-Neto[2]

[1] Division of Endocrinology and Metabolism, Department of Internal Medicine, Universidade de São Paulo, São Paulo – SP, Brazil
[2] Division of Endocrinology and Metabolism, Department of Internal Medicine, Universidade de Brasília, Brasília – DF, CP 4591, CEP 70919-970, Brazil. E-mail: jbn@brnet.com.br

**Abstract**
The inverse relationship between zinc ($Zn^{++}$) and prolactin (PRL) was detected in *in vitro* studies, whereas *in vivo* results are contradictory. In order to evaluate this controversial subject we studied patients with hyperprolactinemia. Basal serum $Zn^{++}$ levels and serum PRL response to acute and chronic oral $Zn^{++}$ administration were evaluated in seven patients with prolactinomas and one with idiopathic hyperprolactinemia. Serum PRL levels did not change after acute oral $Zn^{++}$ administration (37.5 mg), although $Zn^{++}$ levels increased from 1.11 ± 0.15 to 2.44 ± 0.39 µg/mL ($p < 0.05$). $Zn^{++}$ administration (47.7 mg daily) during 60 days increased serum $Zn^{++}$ levels from 1.11 ± 0.15 to 1.59 ± 0.58 µg/mL ($p < 0.05$) but caused no change in serum PRL levels. The TRH tolerance test (200 µg) was performed before and after 60 days of $Zn^{++}$ administration, and PRL response to TRH was unchangeable and similar in both tests. We concluded that acute or chronic $Zn^{++}$ administration does not inhibit PRL secretion in basal condition or by TRH effect in hyperprolactinemic patients.

**Introduction**
$Zn^{++}$ plays an important role in animal and human metabolism, as a constituent or activating co-factor of more than 300 different enzymes. The spectrum of their action includes intermediary metabolism, DNA and RNA synthesis, gene expression, immunocompetence, behaviour, and several endocrine functions [1]. Many studies have shown that $Zn^{++}$ can interact with many hormones especially with PRL [2,3]. Several *in vitro* studies showed that $Zn^{++}$ was capable of inhibiting basal and TRH-stimulated PRL release, in a dose dependent manner, in bovine and rat pituitary cells [4,5,6]. An inverse relationship between serum $Zn^{++}$ and plasma PRL levels is a common finding in human chronic renal failure. The administration of oral $Zn^{++}$ to a group of uremic hypozincemic men decreased their high PRL levels and these levels were inversely correlated to serum $Zn^{++}$ concentrations[7].
In normal male and female subjects and in lambs, acute $Zn^{++}$ administration decreased plasma PRL levels [2,8], although in another report a similar effect was not observed in normal and hyperprolactinemic women [9]. Furthermore, Travaglini et al. described low plasma $Zn^{++}$ levels in eight patients with prolactinoma, which increased to normal levels after bromocriptine-induced PRL normalization [10].
These data point to an inverse relationship between $Zn^{++}$ and PRL, suggesting that this trace element plays an important role in PRL physiology. It was even hypothesized by Koppelman that PRL is a major $Zn^{++}$ regulating hormone and that the suppression of PRL by $Zn^{++}$ is a part of a negative feedback regulatory system [11].
The aim of this paper is to study basal serum $Zn^{++}$ levels, the serum PRL levels after acute and chronic $Zn^{++}$ administration, and TRH-induced PRL secretion after chronic $Zn^{++}$ administration in hyperprolactinemic patients.

**Material and Methods**
*Subjects*
Hyperprolactinemic patients were studied after obtaining written informed consent and approval of the University Ethical Board. The patients examined (seven females and one male) had a mean age of 34 ± 12.3 years (20-52 years) and all but one had radiological evidence of macroadenomas, this one having a normal magnetic resonance image. Patients with ophthalmological and neurological complications and hypopituitarism were excluded.

*Protocol*

The tests were started at 8:00 a.m. at least 12 h after the last meal. A device for infusion was initially inserted into an antecubital vein. All subjects maintained resting decubitus throughout the tests. Blood for $Zn^{++}$ determinations were obtained without tourniquet and all syringes, tubes and pipettes were $Zn^{++}$ free. All samples were stored at –20 ºC.

*TRH Stimulation Test*

Saline infusion was initiated at approximately 8:00 am. Two blood samples for PRL determination were collected 30 min and immediately before an i.v. bolus dose of 200 µg of TRH and after every 15 min during 90 min.

*Acute Oral $Zn^{++}$ Administration*

Blood samples for serum $Zn^{++}$ and PRL determinations were collected 30 min and immediately before 37.5 mg of oral $Zn^{++}$ as $ZnSO_4.7H_2O$ diluted in 20 mL of deionized water and every 30 min during 240 min.

*Chronic Oral $Zn^{++}$ Administration*

All patients received 15.9 mg of elemental zinc, three times a day during 60 days. Basal samples for $Zn^{++}$ and PRL determinations were collected on the 7th, 30th and 60th day. On the 60th day a new TRH stimulation test was performed as described above.

*Biological Analysis*

Serum $Zn^{++}$ was determined in duplicate with an atomic absorption spectrophotometer (Atomspeck model H-1170, Hilger & Watts, UK) as reported [2]. The intra-assay error was 2.5% and sensitivity was 0.02 µg/mL. Hemolyzed samples were discarded. Serum PRL concentrations were measured in duplicate by radioimmunoassay (Diagnostic Products Corporation, USA). The sensitivity of the assay was 1.3 ng/ml and the intra- and interassay coefficients of variations were 6% and 11% respectively.

*Statistical analysis*

All data are given as the mean ± SD. The difference between means was evaluated by the unpaired Student's *t*-test. Tukey's multiple comparison test compared all pairs of columns. Correlation was analyzed according to Spearman's method. Significance was taken as $p < 0.05$ using a significant level of 5%.

### Results

*Basal serum $Zn^{++}$ and serum PRL levels*

Mean basal serum $Zn^{++}$ levels of hyperprolactinemic patients were significantly lower than in normal individuals, 1.11 ± 0.15 and 1.33 ± 0.17 µg/mL, respectively, $p < 0.05$ (Table I). On the other hand, PRL values were higher than in controls, 533 ± 585 and 13.9 ± 3.3 ng/mL, respectively (Table I).

*Acute oral $Zn^{++}$ ingestion*

There was a significant increase of serum $Zn^{++}$ levels during the test when compared with the basal levels, $p < 0.05$ (Table I). Serum PRL did not change as compared to basal levels, not significantly different, *p* (Table I), and no correlation was observed between $Zn^{++}$ and PRL (r = -0.27, $p > 0.05$).

Table I: Acute oral $Zn^{++}$ administration to hyperprolactinemic patients (n=8)

| Time (min) | $Zn^{++}$ (µg/mL) | *p* value [b] | PRL (ng/mL) | *p* value [b] |
|---|---|---|---|---|
| 0 | 1.11 ± 0.15 [a] | | 533 ± 585 [a] | |
| 30 | 1.47 ± 0.44 | > 0.05 | 517 ± 606 | > 0.05 |
| 60 | 1.85 ± 0.53 | > 0.05 | 533 ± 604 | > 0.05 |
| 90 | 2.11 ± 0.53 | < 0.05 | 534 ± 629 | > 0.05 |
| 120 | 2.44 ± 0.39 | < 0.05 | 503 ± 536 | > 0.05 |
| 150 | 2.36 ± 0.58 | < 0.05 | 504 ± 522 | > 0.05 |
| 180 | 2.24 ± 0.71 | < 0.05 | 445 ±476 | > 0.05 |
| 210 | 2.10 ± 0.67 | < 0.05 | 422 ± 456 | > 0.05 |
| 240 | 1.74 ± 0.59 | > 0.05 | 430 ± 460 | > 0.05 |
| Reference range limits [c] | 1.33 ± 0.17 | | 13.99 ± 3.30 | |

[a] Values are expressed as mean ± SD. [b] *P* values were obtained from one-way analysis of variance (ANOVA), using Tukey's multiple comparison test, and $p < 0.05$ was accepted as significant. [c] A reference range study was performed in which serum ($Zn^{++}$) and plasma (PRL) samples from 17 fasting volunteers, including 9 males and 8 females, age-matched, were assayed in our laboratory.

*Chronic oral $Zn^{++}$ ingestion*

Chronic $Zn^{++}$ administration, during 60 days, was followed by an increase of serum $Zn^{++}$ levels as compared to basal values, but they were not significantly difference, $p > 0.05$, and serum PRL levels did not change compared to initial basal values, $p > 0.05$ (Table II). TRH-stimulated PRL on the 60th day of chronic $Zn^{++}$ administration was similar to the test performed before $Zn^{++}$ administration (Table III). Again, no significant correlation was detected between $Zn^{++}$ and PRL (r = -0.25, $p > 0.05$).

Table II: Chronic oral $Zn^{++}$ administration to hyperprolactinemic patients (n=8).

| Days | $Zn^{++}$ (µg/mL) | *p* value [b] | PRL (ng/mL) | *p* value [b] |
|---|---|---|---|---|
| 0 | 1.11 ± 0.15 [a] | | 533 ± 585 [a] | |
| 7 | 1.32 ± 0.15 | > 0.05 | 486 ± 438 | > 0.05 |
| 30 | 1.43 ± 0.30 | > 0.05 | 661 ± 598 | > 0.05 |
| 60 | 1.59 ± 0.58 | > 0.05 | 563 ± 605 | > 0.05 |

[a] Values are expressed as mean ± SD. [b] *P* values were obtained from one-way analysis of variance (ANOVA), using Tukey's multiple comparison test, and $p < 0.05$ was accepted as significant.

Table III: PRL values during TRH stimulation test (200 µg) before and after chronic oral $Zn^{++}$ administration (15.9 mg tid for 60 days) in hyperprolactinemic patients (n=8).

| | PRL (ng/mL) | | *p* value [b] | $Zn^{++}$ (µg/mL) |
|---|---|---|---|---|
| | Basal | Peak | | Basal |
| Before $Zn^{++}$ | 423 ± 439 | 587 ± 572 [a] | > 0.05 | 1.11 ± 0.15 [a] |
| After $Zn^{++}$ | 560 ± 608 | 672 ± 650 | > 0.05 | 1.59 ± 0.58 |
| *p* value [b] | > 0.05 | > 0.05 | > 0.05 | > 0.05 |

[a] Values are expressed as mean ± SD. [b] *P* values were obtained from unpaired *t* test, using two-tail *p* value to compare two groups, and $p < 0.05$ was accepted as significant.

### Discussion

A wide number of *in vitro* studies show an inverse relationship between $Zn^{++}$ and PRL. $Zn^{++}$ interferes physiologically and pharmacologically in the synthesis, storage, release and peripheral action of PRL. Interaction with calcium-channels, calcium-calmodulin complex, adenylate cyclase, secretory granules, membrane stabilization and membrane receptors are some of proposed mechanisms of $Zn^{++}$ involvement in PRL secretion, as was reviewed by Brandão-Neto et al. [1]. In a previous study we have shown that oral $Zn^{++}$ administration to normal male and female individuals induced a significant decrease in PRL levels, suggesting that acute hyperzincemia can inhibit basal PRL secretion [2]. These data support the hypothesis of Koppelman [11] that PRL regulates the uptake and distribution of $Zn^{++}$ and that PRL suppression by this element could represent a negative feedback loop in a similar manner to calcium and PTH.

In this study, our patients with hyperprolactinemia showed lower basal serum $Zn^{++}$ levels (Table I), results similar to that shown by Tavaglini et al. [10]. They are in accordance with the hypothesis of Koppelman [11], although, later, Koppelman et al. [9] were unable to observe the same inverse relationship. The relatively low serum PRL levels of Koppelman's patients as compared with Travaglini's and ours may explain these conflicting results.

In search of this hypothetical loop, we have acutely administered $Zn^{++}$ to hypozincemic hyperprolactinemic patients, which did not result in a decrease of serum PRL levels. Koppelman et al. [9] did not find a serum PRL decrease after $Zn^{++}$ administration to normozincemic hyperprolactinemic patients. Normalization of serum $Zn^{++}$ levels induced by chronic zinc administration to our hyperprolactinemic patients was also not followed by a decrease in serum PRL levels, corroborating data of Travaglini et al. [10]. Before $Zn^{++}$ administration, all patients were submitted to dynamic tests (insulin and GnRH tolerance tests) and no abnormal secretion of GH, LH, FSH, TSH or cortisol was detected.

In humans, acute $Zn^{++}$ administration to normal individuals and hyperprolactinemic patients with normal serum $Zn^{++}$ levels was not followed by a decrease in TRH-stimulated PRL release [9]. In our patients with decreased basal $Zn^{++}$ levels, chronic administration of this trace element did not change the TRH-induced PRL release, despite of a normalization of serum $Zn^{++}$ levels. Initially, we speculated if prolactinoma was out of physiological control but investigations on 15 healthy men submitted to simultaneous $Zn^{++}$ and TRH tolerance tests demonstrated no interference of $Zn^{++}$ on TRH-stimulated PRL secretion [12]. These results practically contradict the hypothesis that the lack of lactotrophs response by $Zn^{++}$ stimulus was a characteristic of autonomy by the prolactinoma, probably due to structural and functional changes in lactotrophs. On the other hand, these data are in conflict with all *in vitro* studies that have shown inhibition of basal and TRH-stimulated PRL release from intact anterior pituitary glands during static incubation and perifused dispersed pituitary cells [3-6]. However, the *in vitro* experiments do not reproduce the intercellular communication by chemical mediators like endocrine, paracrine, synaptic and gap junctions observed *in vivo* [13].

In summary, the present study has shown hypozincemia and no inhibition of TRH-induced PRL secretion during acute and chronic $Zn^{++}$ administration in hyperprolactinemic patients. The fact that zinc administration does not inhibit PRL levels argues against the proposal of using zinc as a therapeutical agent in the treatment of hyperprolactinemic patients.

**Acknowledgements**

This work was partially supported by FAPESP (96/06733-2) and CNPq (# 524130-96).

**References**

[1] J. Brandão-Neto, G. Madureira, B.B. Mendonça, W. Bloise, A.V.B. Castro, *Biol. Trace Elem. Res.* **49** (1995), 139.

[2] J. Brandão-Neto, B.B. Mendonça, T. Shuhama, J.S. Marchini, G. Madureira, M.T.T. Tornero, *Horm. Metabol. Res.* **21** (1989), 203.

[3] M.Y. Lorenson, T. Patel, J.W. Liu, A.M. Walker, *Endocrinolgy* **137** (1996), 809.

[4] F. LaBella, R. Dular, S. Vivian, G. Queen, *Biochem. Biophys. Res. Comm.* **52** (1973), 786.

[5] A.M. Judd, R.M. MacLeod, I.S. Longin, in "The Neurobiology of Zinc", Alan R. Liss, Inc., NY, (1984), pp. 91-104.

[6] R.L. Cooper, J.M. Goldman, G.L. Rehnberg, W.K. McElroy, J.F. Hein, *J. Biochem. Toxicol.* **2** (1987), 241.

[7] S. Mahajan, W. Flamenbaum, R.J. Hamburger, A.S. Prasad, F.D. McDonald, *Lancet* **2** (1985), 750.

[8] D.L., Jr., Rankins, G.S. Smith, D.M. Hallford, *J. Anim. Sci.* **69** (1991), 2932.

[9] M.C.S. Koppelman, V. Greenwood, J. Sohn, P. Deuster, *J. Clin. Endocrinol. Metab.* **68** (1989), 215.

[10] P. Travaglini, E. Mocchegiani, C. De Min, T. Re, N. Fabris, G. Faglia, *J. Neuroscience* **59** (1991), 119.

[11] M.C.S. Koppelman, *Med. Hypotheses* **25** (1988), 65.

[12] A.V.B. Castro, J. Brandão-Neto, B.B. Mendonça, A.L. Mendes, F.J. Campos, in "Metal Ions in Biology and Medicine", John Libbey Eurotext, Paris, (1996), pp. 323-324.

[13] J.F. Habener, in "Williams Textbook of Endocrinology", W.B. Saunders Co., Philadelphia, (1998), pp. 11-41.

**Received: April 2, 1999 - Accepted: May 3, 1999 -**

**Received in revised camera-ready format: May 7, 1999**

